# Ventricle contact may be associated with higher 11C methionine PET uptake in glioblastoma

**DOI:** 10.1007/s00234-021-02742-7

**Published:** 2021-06-10

**Authors:** Bart R. J. van Dijken, Bram Schuuring, Hanne-Rinck Jeltema, Peter Jan van Laar, Roelien H. Enting, Rudi A. J. O. Dierckx, Gilles N. Stormezand, Anouk van der Hoorn

**Affiliations:** 1 Department of Radiology, Medical Imaging Center (MIC), University Medical Center Groningen, P.O. Box 30.001, 9700 RB Groningen, The Netherlands; 2 Department of Neurosurgery, University Medical Center Groningen, Groningen, The Netherlands; 3 Department of Radiology, Hospital Group Twente, Almelo and Hengelo, The Netherlands; 4 Department of Neurology, University Medical Center Groningen, Groningen, The Netherlands; 5 Department of Nuclear Medicine, Medical Imaging Center (MIC), University Medical Center Groningen, Groningen, The Netherlands

**Keywords:** Glioblastoma, 11C Methionine PET, Ventricle contact, Subventricular zone

## Abstract

**Purpose:**

Ventricle contact is associated with a worse prognosis and more aggressive tumor characteristics in glioblastoma (GBM). This is hypothesized to be a result of neural stem cells located around the lateral ventricles, in the subventricular zone. 11C Methionine positron emission tomography (metPET) is an indicator for increased proliferation, as it shows uptake of methionine, an amino acid needed for protein synthesis. This study is the first to study metPET characteristics of GBM in relation to ventricle contact.

**Methods:**

A total of 12 patients with IDH wild-type GBM were included. Using MRI, the following regions were determined: primary tumor (defined as contrast enhancing lesion on T1) and peritumoral edema (defined as edema visible on FLAIR excluding the enhancement). PET parameters in these areas were extracted using PET fused with MRI imaging. Parameters extracted from the PET included maximum and mean tumor-to-normal ratio (TNRmax and TNRmean) and metabolic tumor volume (MTV).

**Results:**

TNRmean of the primary tumor showed significantly higher values for the ventricle-contacting group compared to that for the non-contacting group (4.44 vs 2.67, p = 0.030). Other metPET parameters suggested higher values for the ventricle-contacting group, but these differences did not reach statistical significance.

**Conclusion:**

GBM with ventricle contact demonstrated a higher methionine uptake and might thus have increased proliferation compared with GBM without ventricle contact. This might explain survival differences and should be considered in treatment decisions.

**Supplementary Information:**

The online version contains supplementary material available at 10.1007/s00234-021-02742-7.

## Introduction

Glioblastoma (GBM) is a high-grade primary brain malignancy with a dire prognosis. Despite adequate treatment, median survival ranges from 12 to 18 months [1, 2]. This short life expectancy is related to the almost certain recurrence of GBM, which is due to the infiltrative nature of the tumor [3–5]. Recently, it was demonstrated that GBM with ventricle contact has a worse prognosis when compared to GBM without contact. A meta-analysis showed that ventricle contact is associated with lower survival independent of other prognostic influences [6].

The subventricular zone is an important neural stem cell containing brain region and hypothesized to play a role in the tumorigenesis of GBM [7, 8]. Although ventricle contact has not been shown to be related to a specifically different cell lineage in this regard, it has been associated with more therapy resistance [9]. In addition, ventricle-contacting GBMs have been shown to be more often multifocal and recur further away from the primary tumor, both features being associated with poorer survival [6, 10]. These results seem to suggest that, although a difference in cell of origin has not been demonstrated, ventricle contact is associated with more aggressive tumor behavior. We hypothesized that because of their location, ventricle-contacting GBM could be more prone to stem cell influence and take on more aggressive characteristics, including increased proliferation, than non-contacting GBMs.

MR imaging, the most frequently used imaging modality in studies of GBM, provides several indirect indicators of proliferation, such as tumor size and contrast enhancement. However, neither of these parameters give a direct indication of biological activity of the tumor and are thus limited in appreciating aggressiveness and proliferation.

11C Methionine PET (metPET), a widely employed amino acid tracer, has been shown to indirectly reflect tumor proliferation, as methionine uptake is associated with protein synthesis [11–13]. However, to our best knowledge, studies evaluating metPET characteristics of GBM in relation to ventricle contact have not yet been performed. We hypothesize that a more aggressive behavior of ventricle-contacting GBM will be shown by a higher proliferation indicated by metPET.

## Methods

### Patient population

Data for this study were acquired retrospectively from patient files in the University Medical Centre of Groningen (UMCG) in the Netherlands from 2011 to 2019. The study has been approved by the institutional review board, and the need for written informed consent was waived. We included treatment-naïve patients with isocitrate dehydrogenase wild-type (IDHwt) GBM, following the most recent EANO (European Association of Neuro-Oncology) guidelines [14]. Patients were only eligible for inclusion if they conformed to the following inclusion criteria: (1) IDHwt GBM was confirmed by pathological report, (2) preoperative/prebiopsy metPET imaging was available, and (3) preoperative MRI with at least T1 post-contrast and T2 FLAIR sequences was available. The MRI was required to assess the location of the tumor and to determine the peritumoral area. Exclusion criteria included (1) no contrast enhancing lesion on MRI, (2) other neurological malignant processes, and (3) age below 18. Included patients were first scored on whether the tumors showed lateral ventricle contact by BS and BVD independently, after which an inter-rater agreement score was calculated using a Cohen’s Kappa score [15]. Conflicting scores were settled by AVDH, a neuro-radiologist with more than 5 years of experience in neuro-oncology. Ventricle contact was determined by the extent of the contrast enhancing lesion in relation to the lateral ventricles on the T1 post-contrast sequence on MRI. This was in line with previous studies [16, 17]. The date of diagnosis was defined as the day of the biopsy or operation that confirmed the diagnosis via pathological examination.

### MRI and PET acquisition

Both MRI and PET scans were extracted from the medical files of the included patients. MRI imaging was performed on several different types of machines, from different manufacturers. All MRIs were performed on 1.5 T or 3.0 T. The imaging protocols at least included a 3D post-contrast T1 sequence (repetition time (TR) 2000–2250 ms, echo time (TE) 2.67–3.40 ms, inversion time (TI) 850–900 ms, slice thickness 1 mm, no slice gap, voxel size 1 × 1 × 1 mm) and fluid-attenuated inversion recovery (FLAIR) sequence (TR 5000–11,000 ms, TE 87–337 ms, TI 1800–2800 ms, slice thickness 1–5 mm, no slice gap). Most protocols also included axial T1 pre-contrast, T2, and DWI. Models that were used for scanning included Philips Ingenia, Siemens Aera, Siemens Sonata, and Siemens Avanto. MRI was acquired prior to the PET acquisition. All PET imaging was performed in the UMCG, using either a Siemens Biograph mCT (N = 10) or a PET-HR + (N = 3) scanner (Siemens, Knoxville, TN). Prior to the PET scan, patients fasted for at least 6 h. Static imaging was performed 20 to 40 min after intravenous injection of 11C methionine. For the mCT camera, a low-dose CT was acquired for attenuation correction, and images were reconstructed using Truex + TOF with 3 iterations and 21 subsets in a 400 × 400 matrix size (zoom 1.0). For the HR + camera, a transmission scan was performed immediately after emission scanning in the same bed positions in order to correct for attenuation, and images were reconstructed using OSEM with 3 iterations and 21 subsets. The mean dosage was 205 MBq (range 192 to 224 MBq), radiochemical purity was always higher than 95%, and the specific activity higher than 10,000 GBq/mmol. The 2006 EANM procedure guidelines for brain tumor imaging using labeled amino acid analogues were followed [18].

### Image analysis

**Fig. 1:**
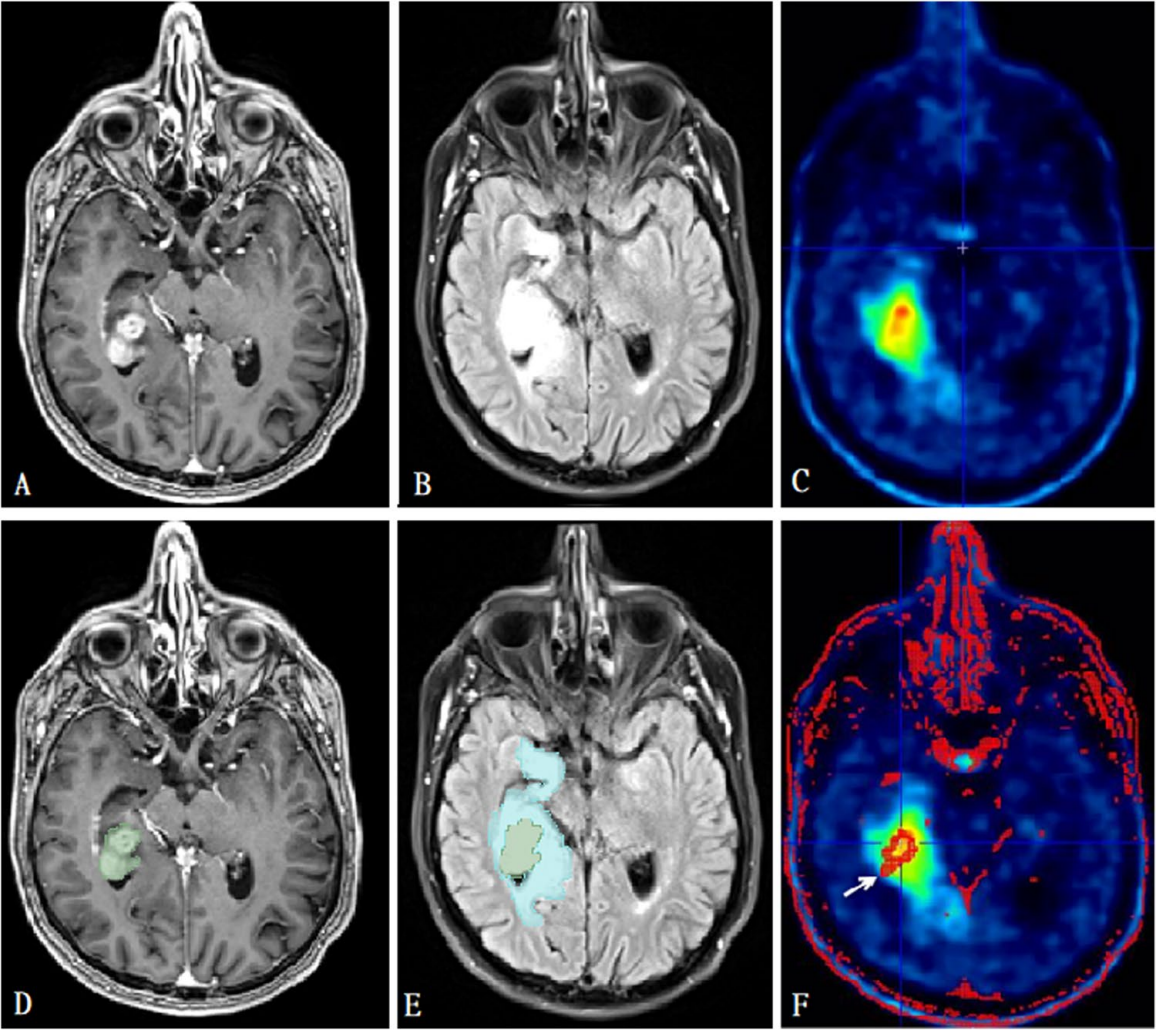
Segmentation of volumes of interest. **A** Post-contrast T1-weighted MRI before segmentation demonstrating a right mesiotemporal ventricle-contacting GBM. **B** FLAIR sequence of the same patient before segmentation. **C** metPET in the same patient before masking with the MRI images demonstrating increased uptake. **D** The same slice as in **A**, but after segmentation of the contrast enhancing lesion (green mask). **E** The same slice as in **B**, but after peritumoral edema segmentation (blue mask). **F** The same slice as in **C**, but after segmentation. The mask based on the contrast enhancing lesion is indicated with an arrow. If necessary, masking was adjusted to match manual segmentations

The different volumes of interest (VOIs), including contrast enhancing tumor and peritumoral edema, were delineated manually. This delineation was performed on the available preoperative MRI using 3DSlicer version 4.10.2 (http://www.slicer.org). The primary tumor was delineated as the contrast enhancing area on T1; internal cystic or necrotic tissue was not excluded from the primary tumor VOI. Peritumoral area was determined using the T2 FLAIR sequence; it was defined as the area of high intensity surrounding the tumor, excluding the primary tumor VOI as already determined on the post-contrast T1-weighted sequence. These delineations were made manually on a slice by slice basis as illustrated in Fig. 1A and B. Besides these sequences, unenhanced T1 and T2 were also used to support assessment. The determined VOIs were then used as a supporting tool in PET analysis using PMOD version 4.1 (PMOD Technologies, Zurich, Switzerland). PET scans were co-registered to corresponding MRI, making it possible to use the MRI for overlays on the PET. Using the semiautomatic delineation tools in PMOD, the primary tumor as visible on T1 post-contrast-weighted MRI was converted into a mask, which could be overlaid on the PET scan (Fig. 1C & D). These VOIs were compared to the predetermined delineations of the 3DSlicer and adjusted where necessary. Additionally, the high tracer uptake PET lesion was also delineated semi automatically using the PMOD software. Forty percent of the tumor SUVmax was used as a threshold to determine the high PET uptake lesion. The mask was used to divide the high PET uptake lesion into two parts: (1) the high PET uptake lesion inside the mask and (2) the high PET uptake lesion outside the mask. The combination of the 3DSlicer and PMOD was thus used because 3DSlicer allowed for a more reliable tumor delineation on MRI, while PMOD allowed for the extraction and calculation of PET parameters used in our analysis.

### Imaging parameters

PET parameters were collected from these VOIs separately, where parameters from inside the mask were defined as those of the primary tumor, and parameters from outside the mask were defined as those of the peritumoral area. Several PET parameters were determined within these two VOIs and included maximum and mean tumor-to-normal-ratios (TNRmax and TNRmean, respectively) as metabolic parameters and metabolic tumor volume (MTV) as the volumetric parameter. For each voxel, the standardized uptake value (SUV) was determined as follows.

SUV = C(T)/[injection dose(MBq)/patient weight (kg)] [19]

The TNR values were calculated by dividing the corresponding SUV within the VOI by the SUV within a 1-cm radius sphere mirrored on the contralateral centrum semiovale. MTV represents the volume of the PET-enhancing area in milliliter.

Additionally, the diameter of the contrast enhancing tumor and the volumes of the primary tumor and the peritumoral edema as defined by the delineations made in 3Dslicer were collected.

### Statistics

All statistical analysis was performed in SPSS version 23.0 (Armonk, NY, IBM Corp.). Patient characteristics were compared using the corresponding non-parametric statistical tests. Proportions were compared using a chi square test and medians were compared with a Mann–Whitney U test. Median values of the PET parameters and tumor volumes were determined, and differences in rankings were tested using the Mann–Whitney U test. Statical significance was set at a two-sided p-value of 0.05 throughout.

## Results

### Patient population

The 12 included patients had a median age of 59 years (range 41–73). No significant differences were observed between the two groups. The characteristics and associated significance values are shown in Table 1.

**Table 1:** Comparing patient characteristics of the ventricle-contacting group with the non-contacting group

	Ventricle contact		
	No	Yes	Sig
Median age at diagnosis in years (range)	61 (47–73)	52 (41–63)	0.222
Sex (N)			
Female	2	2	0.679
Male	5	3	
Resection (N)			
Gross total	3	2	0.152
Subtotal	0	2	
Biopsy	4	1	
Use of corticosteroids at time of metPET (N)			
Yes	1	3	0.098
No	6	2	
MGMT methylation status			
Methylated	4	1	0.198
Unmethylated	-	-	
Missing	3	4	
Median time between PET and MRI in days (range)	9 (4–23)	10 (0–29)	1.000

Table showing patient characteristics and comparing the ventricle-contacting group with the non-contacting group. Median scores were tested with a Mann–Whitney U test, while all other scores were tested using a Chi square test

After scoring these patients for ventricle contact, there was only one disagreement resulting in a good inter-rater agreement with a kappa of 0.80 (20). Of the 12 included patients, 5 were found to have a ventricle-contacting tumor (38.5%), while 7 had a non-contacting tumor (61.5%). There was no difference in volumes of contrast enhancing lesion or FLAIR hyperintense area between ventricle-contacting tumors and non-contacting tumors, although the maximum diameter of ventricle-contacting tumors was larger than non-contacting tumors, as shown in Supplementary Table 1.

### 11C Methionine PET parameters

All PET parameters of both the primary tumor VOI and the peritumoral VOI demonstrated higher median values for the ventricle-contacting group compared to the non-contacting group, as well as higher mean ranks (Table 2). Higher metabolism was shown within the primary tumor VOI with a statistically significant higher TNRmean of 4.44 (range 2.94–7.74) for the ventricle-contacting group versus 2.67 (range 1.32–3.44) for the non-contacting group, p = 0.030. For TNRmax and MTV, the differences between the ventricle-contacting and non-contacting groups were non-significant (p = 0.106). MTV was not significantly different between. Despite that all peritumoral VOI parameters were higher in the ventricle-contacting group, none of the differences showed statistical significance, with p-values ranging from 0.148 to 0.202.

**Table 2:** PET parameters of primary tumor and peritumoral volumes of interest

				Mann–Whitney U test results			
	Ventricle contact	N	Median score (range)	Mean rank	Sum of ranks	Z value	Sig
TNRmax Primary	Yes	5	4.70 (2.57–6.17)	8.60	43	− 1.705	0.106
	No	7	2.70 (1.72–3.22)	5.00	35		
TNRmean Primary	Yes	5	4.44 (2.94–7.74)	9.20	46	− 2.192	**0.030**
	No	7	2.67 (1.32–3.44)	4.57	32		
MTV (cm^3^) Primary	Yes	5	6.75 (0.22–13.83)	8.60	43	− 1.705	0.106
	No	7	0.39 (0.05–3.38)	5.00	35		
TNRmax Peritumoral	Yes	5	5.08 (1.96–6.13)	8.20	41	− 1.380	0.202
	No	7	2.70 (1.94–3.29)	5.29	37		
TNRmean Peritumoral	Yes	5	4.62 (2.48–5.38)	8.40	42	− 1.543	0.148
	No	7	2.91 (1.35–3.60)	5.14	36		
MTV (cm^3^) Peritumoral	Yes	5	10.84 (4.39–28.18	8.20	41	− 1.380	0.202
	No	7	3.06 (0.68–36.14)	5.29	37		

Table showing the median scores and Mann–Whitney U test results of the PET parameters of the primary tumor area as determined by T1 post-contrast MRI and the peritumoral area as determined by FLAIR. Abbreviations: *TNR* tumor-to-normal ratio, *MTV* metabolic tumor volume, *Primary* primary tumor, *Peritumoral* peritumoral area

The boldface value (0.030) is actually significant (<0.05)

## Discussion

This is the first study comparing metPET characteristics of GBM in relation to the ventricle contact. Our results show an increased methionine uptake for ventricle-contacting GBMs in comparison to that for non-contacting GBMs. Specifically, our results demonstrated significantly higher TNRmean for ventricle-contacting tumors than that for non-contacting tumors. This would imply an increased protein synthesis and therefore higher proliferation in the ventricle-contacting group, further supporting the more aggressive characteristics of ventricle-contacting GBMs.

Ventricle contact in GBM has previously been associated with several imaging characteristics related to tumor aggressiveness, such as larger tumor volumes, more multifocal and distant recurrences, and higher peritumoral perfusion, all of which potentially contribute to the lower survival of these tumors [6, 9, 10]. In addition to this, we have now also demonstrated a higher methionine uptake, as a measure of increased proliferation, for ventricle-contacting GBM.

Although our study is the first to study metPET in relation to ventricle contact in GBM, our results are in line with a recent study looking at cellular proliferation with *O*-(2-[18F]fluoroethyl)-l-tyrosine (FET) PET [20]. Although it was not the primary research question, FET uptake showed a significantly higher SUVmean value for GBM with ventricle involvement when compared to that for GBM without ventricle contact.

We also hypothesized that ventricle contact in GBM could be related to higher methionine uptake levels in the peritumoral area. The peritumoral area plays an important role in the recurrence of GBM as it contains both vasogenic edema and infiltrating tumor cells. The peritumoral area is of particular interest in relation to ventricle contact in GBM as one characteristic of ventricle-contacting GBM is a more distant and multifocal recurrence [6, 10].

In addition, a recent study showed a higher peritumoral perfusion for ventricle-contacting GBMs, associated with a more aggressive peritumoral infiltration [21]. In line with these hypotheses, we did find higher values for metPET parameters in the peritumoral region, but none of those differences reached significance.

Despite the significant difference in TNRmean between ventricle-contacting and non-contacting GBMs, this study does have its limitations. The first limitation is the small sample size. This fact is a direct cause for the inability to meet assumptions for normal distribution and asymptotic significance, as well as a limited power of the outcomes. It is because of this that this study should primarily be seen as a pilot study and an invitation to perform larger prospective studies. Nevertheless, a significant difference for TNRmean has been observed, even with this limited sample size. Furthermore, the other PET parameters point in the same direction although not reaching significance. A second limitation is the retrospective nature of our data, resulting in the lack of a standardized protocol, such as the use of two PET systems with different resolutions. These different resolutions could potentially have introduced partial volume effects. However, by employing TNR and thus dividing uptake values by the background uptake, we aimed to limit these partial volume effects. Moreover, standard phantom studies were performed upon acceptance of both scanners to ensure the quality of both camera systems. The lack of standardization also resulted in a heterogeneous time interval between PET and MRI acquisition, which in some cases amounted to as much as 29 days. Additionally, due to the retrospective nature, MGMT methylation status was not available for all patients in this study. Prospective studies should make sure that time between MRI and PET is minimalized, which would lead to a smaller inaccuracy when performing the MRI overlay on the PET. Finally, it should be addressed that there seems to be a non-significant difference between the two groups regarding corticosteroid use. Corticosteroids potentially influence imaging characteristics, most notably the FLAIR hyperintense area. However, there was no difference in hyperintense FLAIR volume between the two groups. Furthermore, contrast enhancement (the primary tumor VOI) is not affected by corticosteroid use.

## Conclusion

Ventricle-contacting GBMs demonstrated a significantly higher uptake of methionine than non-contacting tumors. The increased uptake of methionine indirectly indicates a higher proliferation. These findings possibly explain the survival difference between ventricle-contacting and non-contacting GBMs and should possibly be considered in treatment decisions. Larger prospective studies are necessary to confirm our observations.

## Supplementary Information

Below is the link to the electronic supplementary material.

ESM 1 (DOCX 14 kb)
