# Supplementary material for: Ventricle contact may be associated with higher 11C methionine PET uptake in glioblastoma
Source: Neuroradiology. 2021 Jun 10;64(2):247–52. doi: 10.1007/s00234-021-02742-7 (PMC8789691; doi:10.1007/s00234-021-02742-7)
Supplement: Supplementary file 1 — (DOCX 14 kb) [file 234_2021_2742_MOESM1_ESM.docx]

**ONLINE SUPPLEMENTARY MATERIAL**

**Supplementary table 1 – MRI parameters**

|  |  |  |  |  |
| --- | --- | --- | --- | --- |
|  | Ventricle contact | n | Mean (SD) |  |
|  |  |  |  | Sig. |
| Volume of contrast enhancing lesion (cm^3^) | Yes | 5 | 19.32 (15.48) | 0.082 |
|  | No | 7 | 3.40 (4.34) |  |
| Volume of FLAIR hyperintense area (cm^3^) | Yes | 5 | 61.31 (46.61) | 0.759 |
|  | No | 7 | 71.19 (62.14) |  |
| Maximum tumor diameter (cm) | Yes | 5 | 4.96 (1.10) | **0.004** |
|  | No | 7 | 2.46 (0.83) |  |
